# Bedaquiline resistance in drug-resistant tuberculosis HIV co-infected patients

**DOI:** 10.1183/13993003.02383-2019

**Published:** 2020-06-04

**Authors:** Camus Nimmo, James Millard, Kayleen Brien, Sashen Moodley, Lucy van Dorp, Keeren Lutchminarain, Allison Wolf, Alison D. Grant, Francois Balloux, Alexander S. Pym, Nesri Padayatchi, Max O'Donnell

**Affiliations:** 1 Division of Infection and Immunity, University College London, London, UK; 2 UCL Genetics Institute, University College London, London, UK; 3 Africa Health Research Institute, Durban, South Africa; 4 Wellcome Trust Liverpool Glasgow Centre for Global Health Research, Liverpool, UK; 5 Institute of Infection and Global Health, University of Liverpool, Liverpool, UK; 6 National Health Laboratory Service, Durban, South Africa; 7 Dept of Medicine, Columbia University Medical Center, New York, NY, USA; 8 TB Centre, London School of Hygiene and Tropical Medicine, London, UK; 9 CAPRISA MRC-HIV-TB Pathogenesis and Treatment Research Unit, Durban, South Africa; 10 Dept of Medicine and Epidemiology, Columbia University Medical Center, New York, NY, USA

## Abstract

Global tuberculosis (TB) control is threatened by drug resistance, with over 500 000 cases resistant to first-line drugs in 2018 [1]. Bedaquiline is a highly effective TB drug and has improved drug-resistant TB (DR-TB) outcomes in trial and programmatic settings [2, 3]. The World Health Organization (WHO) recommends its inclusion in most DR-TB regimens [4] and it is under further evaluation in clinical trials. There have been several reports of clinical bedaquiline resistance [5–8]. Resistance-associated variants (RAVs) in clinical isolates identified to date are almost exclusively caused by *Rv0678* mutations which can raise *Mycobacterium tuberculosis* minimum inhibitory concentrations (MICs) for bedaquiline and clofazimine [9].

## Abstract 2

**Genetic mutations linked to bedaquiline resistance were found before starting treatment and acquired during treatment in patients with drug-resistant TB and HIV in KwaZulu-Natal, South Africa. Routine bedaquiline resistance testing needs to be accelerated.** http://bit.ly/2vnL4VY

*To the Editor*:

Global tuberculosis (TB) control is threatened by drug resistance, with over 500 000 cases resistant to first-line drugs in 2018 [1]. Bedaquiline is a highly effective TB drug and has improved drug-resistant TB (DR-TB) outcomes in trial and programmatic settings [2, 3]. The World Health Organization (WHO) recommends its inclusion in most DR-TB regimens [4] and it is under further evaluation in clinical trials. There have been several reports of clinical bedaquiline resistance [5–8]. Resistance-associated variants (RAVs) in clinical isolates identified to date are almost exclusively caused by *Rv0678* mutations which can raise *Mycobacterium tuberculosis* minimum inhibitory concentrations (MICs) for bedaquiline and clofazimine [9].

The South African province of KwaZulu-Natal was the site of an extensively drug-resistant TB (XDR-TB) outbreak among HIV co-infected patients [10, 11]. Despite extensive bedaquiline use in KwaZulu-Natal, routine phenotypic or genotypic drug susceptibility testing (DST) is not performed, leaving the potential for unidentified bedaquiline resistance to spread.

The clinical significance of baseline *Rv0678* variants is unclear [12]. Emergence of *Rv0678* mutations during treatment has been documented but their incidence is unknown. We report the frequency of baseline and emergent bedaquiline RAVs and associated clinical outcomes in a prospective DR-TB cohort treated with bedaquiline and clofazimine.

Adult patients with DR-TB and HIV presenting at a public TB referral hospital in KwaZulu-Natal, South Africa were enrolled within two weeks of starting bedaquiline in the PRAXIS study (NCT03162107) between November 2016 and January 2019. Treatment with antiretroviral therapy was a mandatory inclusion criterion. Clinical data, questionnaires, and sputum were collected at monthly visits over the first 6 months of treatment, with end of treatment follow-up. The study was approved by the University of KwaZulu-Natal Biomedical and the Columbia University ethics review boards. All sputa were inoculated into mycobacterial growth indicator tubes and cultured in a BACTEC 960 (BD, MD, USA). Positive cultures underwent whole genome sequencing (WGS) and bedaquiline MIC testing was performed for isolates with *Rv0678* variants using the proportion method on 7H11 agar. Culture conversion at 6 months was defined as two or more consecutive negative monthly cultures. Outcomes at the end of treatment were assigned according to standard definitions [13].

Of 965 adult TB patients who presented during the study period, 297 were eligible to participate and consented to enrolment. The most common reasons for ineligibility were not being HIV co-infected (n=160) and starting bedaquiline >2 weeks previously (n=126). Positive baseline cultures and WGS results were available for 92 patients who are the subjects of this report. The remaining 205 patients were culture negative at baseline (n=198) or had isolates that failed WGS (n=7). The sequenced cohort was 51% female and median age was 36 years (interquartile range (IQR) 30–43 years); 66% had a previous history of any TB and 23% of DR-TB. The median CD4 count was 276 (IQR 134–452). Patients with sequenced isolates were more likely to have second-line drug resistance (53/92; 57.6% *versus* 82/205; 40.0%; p=0.006) but otherwise had similar baseline characteristics.

Baseline *Rv0678* variants were identified in 5.4% (5/92) patients with a sequenced positive baseline culture prior to initiating bedaquiline treatment (figure 1). Although none of the MICs for samples with baseline *Rv0678* variants exceeded the bedaquiline critical concentration, 3/5 had MICs at the top of the wild-type range (0.25 µg·mL^−1^). The bedaquiline MIC of patient B, who had a baseline *Rv0678* variant, increased from 0.03 µg·mL^−1^ at baseline to 0.25 µg·mL^−1^ at month 2 with an increase in *Rv0678* variant allele frequency from 72% to 96%. Additional emergent *Rv0678* variants occurred in 5.7% (5/87) patients during treatment (figure 1). Emergent *Rv0678* variants were associated with a >8-fold increase in bedaquiline MIC.

**FIGURE 1:**
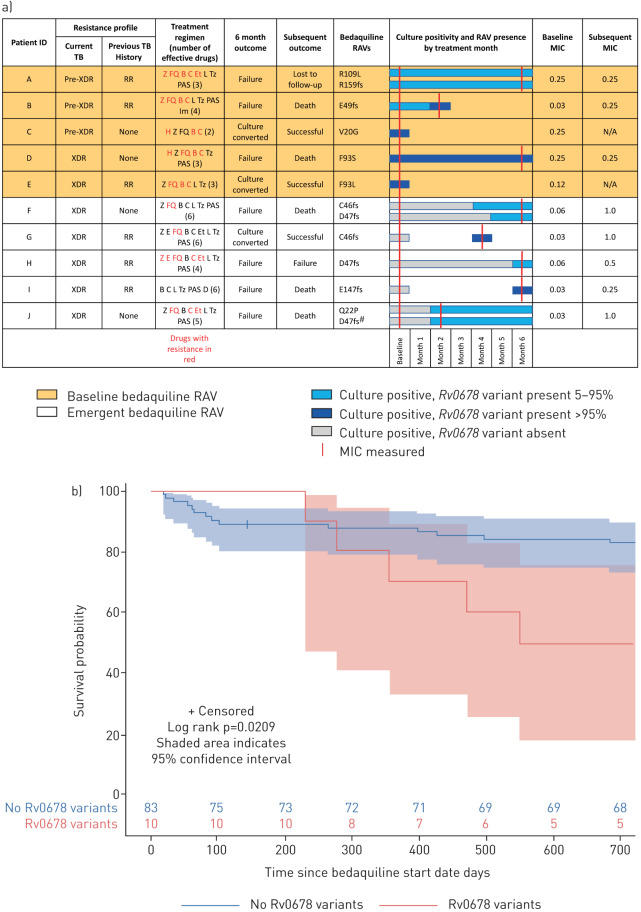
a) Patients with *Rv0678* mutations in positive tuberculosis (TB) sputum cultures. Resistance profiles are for the current and most recent previous TB episodes. Drugs used in treatment regimen are indicated, with those ineffective due to resistance coloured red. H: isoniazid; Z: pyrazinamide; E: ethambutol; FQ: fluoroquinolones; B: bedaquiline; C: clofazimine; Et: ethionamide; L: linezolid; T: terizidone; PAS: p-aminosalicylic acid; D: delamanid; Im: imipenem; RAV: resistance-associated variant; XDR: extensively drug resistant; RR: rifampicin resistant. Patient A was phenotypically ethionamide resistant in the absence of ethionamide resistance-associated variants. *Rv0678* variants are categorised as baseline (orange background) or emergent (white background). Amino acid changes at variant sites are specified (fs, frameshift mutation). Bars indicate culture-positive samples without variants (grey), heterozygous variants (light blue) and fixed variants (dark blue). Minimum inhibitory concentrations (MICs) are shown at baseline and at subsequent time-point if performed (red lines). ^#^: in patient J, six further low-frequency *Rv0678* variants appeared at 6 months (A57E, R72 T, D88fs, D88A, G121R, L122P). b) Kaplan–Meier curve for survival probability following initiation of bedaquiline therapy with censoring for loss to follow-up. Shaded area indicates 95% confidence interval.

All patients with baseline or emergent *Rv0678* variants had resistance to fluoroquinolones or second-line injectables (pre-XDR-TB/XDR-TB) and 60% had previously been treated for DR-TB. Patients with *Rv0678* mutations were more likely to have XDR-TB (60%) than those without (29%) (p=0.07). None were previously treated with bedaquiline or clofazimine. No *pepQ* or *atpE* mutations were identified. No further phenotypic or genotypic resistance to drugs other than bedaquiline/clofazimine emerged during treatment. Baseline drug resistance profiles and regimens for patients with *Rv0678* variants are shown (figure 1).

In 4/5 cases, emergent bedaquiline resistance occurred due to within-patient evolution of the infecting strain, while in one case (patient G) resistance was suggestive of superinfection with a bedaquiline-resistant strain from patient F. Both patients were hospitalised during the same period and this appears to represent nosocomial transmission as there was no evidence of cross-contamination.

Median follow-up duration for the entire cohort was 12.8 months (IQR 6.0–18.9) and mortality was 20.7% (19/92). Overall, 73/92 (79.3%) patients culture converted by 6 months. Among patients without *Rv0678* mutations, 70/82 (85%) culture converted, while in patients with baseline *Rv0678* mutations 2/5 (40%) culture converted. Among five patients with baseline *Rv0678* variants, 3/5 (60%) had an unsuccessful outcome (two deaths and one loss to follow-up) compared to 16/82 (18.4%) in patients without *Rv0678* variants (p=0.058). Patients with emergent *Rv0678* variants were more likely to be culture-positive at 6 months than those without (4/5; 80.0% *versus* 12/82; 14.6%; p=0.004) and 4/5 (80.0%) died or were lost to follow-up compared to 15/82 (18.3%) without (p=0.007). Among patients with a baseline or emergent *Rv0678* variant, 70% (7/10) had an unsuccessful outcome (death, treatment failure, or loss to follow-up) compared to 18% (15/82) without (p=0.001).

The distribution of MICs in patients with *Rv0678* variants in this study are consistent with other reports, finding that many variants are associated with raised MICs below or at the critical concentration [14]. All isolates with MICs >0.25 µg·mL^−1^ in this study had C46 or D47 frameshift mutations and were only seen in acquired drug resistance. Strains with higher (but technically susceptible) MICs for other TB drugs have also been linked to a higher risk of relapse [15]. The key question is whether bedaquiline MICs at or just below the critical concentration of 0.25 µg·mL^−1^ (on 7H11 agar) have clinical consequences and undermine guidelines on bedaquiline phenotypic DST for clinical decision making and monitoring resistance transmission.

It is concerning that all five patients with emergent resistance had four or more active drugs in their treatment regimen. Interestingly, no other emergent resistance was found during follow-up. Bedaquiline may represent such a key drug within treatment regimens that resistance develops either to bedaquiline or to none at all, and due to its long half-life, resistance may occur as a result of prolonged exposure to subtherapeutic levels when adherence is suboptimal. The combination of bedaquiline and clofazimine may also have contributed to selection of resistance as *Rv0678* mutations confer cross-resistance and all patients received a combination of both drugs [9].

The percentage of baseline *Rv0678* variants identified in our study was similar to the 6.6% identified in the C208 and C209 bedaquiline clinical trials [8]. C209 reported >4-fold MIC increases associated with *Rv0678* variants in 12/205 (4.4%) patients, similar to the 4/92 (4.3%) in our study, but no association with outcome [12]. Presence of baseline *Rv0678* mutations may indicate current transmission of bedaquiline and clofazimine resistant strains in the community.

Limitations of this study include the relatively small number of patients evaluable. The number of patients with *Rv0678* variants was also small, limiting causal interpretation of clinical outcome differences. While South Africa is an important early adopter of bedaquiline, the high HIV prevalence and unique TB epidemic may limit generalisation of our findings. Factors related to poor outcome tend to cluster (*e.g.* low medication adherence, suboptimal HIV control and substance abuse), making it difficult to disentangle to what extent bedaquiline resistance is causative of poor outcome or merely a co-variate. Strengths of this study include its prospective design, longitudinal follow-up, and carefully collected clinical outcomes.

This study identifies an important subpopulation of DR-TB HIV patients with baseline and emergent bedaquiline RAVs associated with poor clinical outcomes. We highlight a role for active genotypic monitoring to identify bedaquiline resistance, as well as re-evaluation of phenotypic DST critical concentrations. This report also raises concerns surrounding the overall strategy of empiric treatment regimens for DR-TB, even when constructed with novel agents, and suggests individualised treatment regimens guided by sequencing may be required to achieve optimal treatment outcomes in all patients and prevent the emergence of bedaquiline resistant DR-TB strains.

## Shareable PDF

10.1183/13993003.02383-2019.Shareable1 This one-page PDF can be shared freely online.
Shareable PDF ERJ-02383-2019.Shareable

## Supplementary Material

ERJ-02383-2019.Shareable.pdf
