# Supplementary material for: Bedaquiline resistance in drug-resistant tuberculosis HIV co-infected patients
Source: Eur Respir J. 2020 Jun 4;55(6):1902383. doi: 10.1183/13993003.02383-2019 (PMC7270361; doi:10.1183/13993003.02383-2019)
Supplement: Supplementary file 1 [file ERJ-02383-2019.Shareable.pdf]

SHAREABLE PDF

Bedaquiline resistance in drug-resistant tuberculosis HIV co-infected patients

Camus Nimmo [1,2,3], James Millard [3,4,5], Kayleen Brien [3], Sashen Moodley [3], Lucy van Dorp [2], Keeren Lutchminarain [6], Allison Wolf [7], Alison D. Grant [3,8], Francois Balloux [2], Alexander S. Pym [3], Nesri Padayatchi [9] and Max O'Donnell [9,10]

Affiliations: [1]Division of Infection and Immunity, University College London, London, UK. [2]UCL Genetics Institute, University College London, London, UK. [3]Africa Health Research Institute, Durban, South Africa. [4]Wellcome Trust Liverpool Glasgow Centre for Global Health Research, Liverpool, UK. [5]Institute of Infection and Global Health, University of Liverpool, Liverpool, UK. [6]National Health Laboratory Service, Durban, South Africa. [7]Dept of Medicine, Columbia University Medical Center, New York, NY, USA. [8]TB Centre, London School of Hygiene and Tropical Medicine, London, UK. [9]CAPRISA MRC-HIV-TB Pathogenesis and Treatment Research Unit, Durban, South Africa. [10]Dept of Medicine and Epidemiology, Columbia University Medical Center, New York, NY, USA.

Correspondence: Max O'Donnell, Dept of Medicine and Epidemiology, Columbia University Medical Center, New York, NY, USA. E-mail: mo2130@cumc.columbia.edu

@ERSpublications
Genetic mutations linked to bedaquiline resistance were found before starting treatment and acquired during treatment in patients with drug-resistant TB and HIV in KwaZulu-Natal, South Africa. Routine bedaquiline resistance testing needs to be accelerated. http://bit.ly/2vnL4VY

Cite this article as: Nimmo C, Millard J, Brien K, *et al.* Bedaquiline resistance in drug-resistant tuberculosis HIV co-infected patients. *Eur Respir J* 2020; 55: 1902383 [https://doi.org/10.1183/13993003.02383-2019].

This single-page version can be shared freely online.

To the Editor:

Global tuberculosis (TB) control is threatened by drug resistance, with over 500 000 cases resistant to first-line drugs in 2018 [1]. Bedaquiline is a highly effective TB drug and has improved drug-resistant TB (DR-TB) outcomes in trial and programmatic settings [2, 3]. The World Health Organization (WHO) recommends its inclusion in most DR-TB regimens [4] and it is under further evaluation in clinical trials. There have been several reports of clinical bedaquiline resistance [5–8]. Resistance-associated variants (RAVs) in clinical isolates identified to date are almost exclusively caused by *Rv0678* mutations which can raise *Mycobacterium tuberculosis* minimum inhibitory concentrations (MICs) for bedaquiline and clofazimine [9].

Copyright ©ERS 2020.. This version is distributed under the terms of the Creative Commons Attribution Licence 4.0.
